# 

**Published:** 1801-01

**Authors:** 


### MEDICAL AND PHYSICAL JOURNAL,

CONTAINING THE

EARLIEST INFORMATION ON SUBJECTS

OF

MEDICINE, SURGERY, PHARMACY, CHEMISTRY,

AND

NATURAL HISTORY;

AND A

CRITICAL ANALYSIS OF ALL NEW BOOKS

IN THOSE

DEPARTMENTS OF LITERATURE.

CONDUCTED BY

T. BRADLEY, M. D.

Member of the Royal College of Physicians, London; Physician to the Westminster Hospital, and to the Asylum for Female Orphans; Lecturer on the Theory and Practice of Medicine, &c.

BY

R. BATTY, M. D.

Licentiate in Midwifery, of the Royal College of Physicians, London; of the British Lying-In-Hospital; Physician to the Infant Asylum; Fellow of the Linnean Society; and Lecturer on Midwifery.

AND BY

A. A. NOEHDEN, M. D.

Vice-Secretary of the Physical Society of Gottingen; Corresponding Member of the Linnean Society, London, and of the Medical Society at Paris.

VOL. V.

FROM JANUARY TO JUNE, 1801.

Surgeon Gen'l's Office LIBRARY. Washington, D.C. 5111

LONDON:

Printed for R. PHILLIPS, No. 71, St. Paul's Church-Yard: and sold by all Booksellers in the United Kingdoms.

*Price Twelve Shillings in Boards.*

[J. D. Dewick, Printer, Aldersgate Street.]

### TO CORRESPONDENTS.

*Communications are received from Messrs. Custance, Kynion, Jones, Macgarth, Bartley, Spry, Simmons, and Hill; and from Drs. De Carro, Mossman, and Woodforde, which have been unavoidably postponed for want of room.*

*The great increase of original Communications has obliged us to defer an account of several valuable Books, and the Foreign Literature.*

